# Long chain capsaicin analogues synthetized by CALB-CLEAs show cytotoxicity on glioblastoma cell lines

**DOI:** 10.1007/s00253-023-12856-y

**Published:** 2024-01-12

**Authors:** Tania Diaz-Vidal, Vicente Paúl Armenta-Pérez, Luis Carlos Rosales-Rivera, Georgina Cristina Basulto-Padilla, Raúl Balam Martínez-Pérez, Juan Carlos Mateos-Díaz, Yanet K. Gutiérrez-Mercado, Alejandro A. Canales-Aguirre, Jorge A. Rodríguez

**Affiliations:** 1 Biotecnología Industrial, Centro de Investigación y Asistencia en Tecnología y Diseño del Estado de Jalisco, CIATEJ, 45019 Zapopan, Mexico; 2 Departamento de Ingeniería Química, CUCEI, Universidad de Guadalajara, 44430 Guadalajara, Mexico; 3 Present Address: Departamento de Biotecnología y Ciencias Alimentarias, Instituto Tecnológico de Sonora, 85137 Ciudad Obregón, Mexico; 4 Unidad de Evaluación Preclínica, Unidad de Biotecnología Médica y Farmacéutica, Centro de Investigación y Asistencia en Tecnología y Diseño del Estado de Jalisco, CIATEJ, 44270 Guadalajara, Mexico; 5 Present Address: Laboratorio Biotecnológico de Investigación y Diagnóstico, Departamento de Clínicas, División de Ciencias Biomédicas, Centro Universitario de los Altos, Universidad de Guadalajara, Tepatitlán de Morelos, Jalisco Mexico

**Keywords:** Cross-linked enzyme aggregates, Capsaicin analogues, *Candida antarctica* lipase B, Punivanil, Punicic acid, Glioblastoma

## Abstract

**Abstract:**

Glioblastoma is one of the most lethal tumors, displaying striking cellular heterogeneity and drug resistance. The prognosis of patients suffering from glioblastoma after 5 years is only 5%. In the present work, capsaicin analogues bearing modifications on the acyl chain with long-chain fatty acids showed promising anti-tumoral activity by its cytotoxicity on U-87 and U-138 glioblastoma multiforme cells. The capsaicin analogues were enzymatically synthetized with cross-linked enzyme aggregates of lipase B from *Candida antarctica *(CALB). The catalytic performance of recombinant CALB-CLEAs was compared to their immobilized form on a hydrophobic support. After 72 h of reaction, the synthesis of capsaicin analogues from linoleic acid, docosahexaenoic acid, and punicic acid achieved a maximum conversion of 69.7, 8.3 and 30.3% with CALB-CLEAs, respectively. Similar values were obtained with commercial CALB, with conversion yields of 58.3, 24.2 and 22% for capsaicin analogues from linoleic acid, DHA and punicic acid, respectively. Olvanil and dohevanil had a significant cytotoxic effect on both U-87 and U-138 glioblastoma cells. Irrespective of the immobilization form, CALB is an efficient biocatalyst for the synthesis of anti-tumoral capsaicin derivatives.

**Key points:**

*• This is the first report concerning the enzymatic synthesis of capsaicin analogues from docosahexaenoic acid and punicic acid with CALB-CLEAs.*

*• The viability U-87 and U-138 glioblastoma cells was significantly affected after incubation with olvanil and dohevanil.*

*• Capsaicin analogues from fatty acids obtained by CALB-CLEAs are promising candidates for therapeutic use as cytotoxic agents in glioblastoma cancer cells.*

**Supplementary Information:**

The online version contains supplementary material available at 10.1007/s00253-023-12856-y.

## Introduction

The broad applicability of capsaicin (8-methyl-*N*-vanillyl-6-nonenamide) and capsaicin analogues in the food, pharmacy, and health industry has led to a renewed interest in these compounds (Abdel-Salam 2014; Basith et al. 2016; Santos et al. 2023). Among the spectrum of applications, perhaps the most promising one is the apoptotic effect of capsaicin in several human cancer cell lines such as KB (Lin et al. 2013), prostate cancer (Mori et al. 2006), and glioblastoma cells (Lee et al. 2000; Szoka and Palka 2020). Glioblastomas account for 46% of primary malignant brain tumors, and the survival rate of patients diagnosed with this form of glioma is only 5% (Ostrom et al. 2015). Capsaicin induces apoptosis in several human glioblastoma cells, such as A172 (Lee et al. 2000), LN-18 (Szoka and Palka 2020), U87-MG (Jeon et al. 2012), and U373 (Amantini et al. 2007), in a dose-dependent manner. The apoptotic induction of capsaicin is linked to an up-regulation of PPARɣ and an activation of caspases-9 and -3, and it is not related with a stimulatory effect on vanilloid receptors nor with an increase in intracellular calcium ions (Lee et al. 2000; Jacobsson et al. 2001; Szoka and Palka 2020).

Besides the natural capsaicinoids present in *Capsicum* plants, several capsaicin analogues have been synthetically obtained at laboratory scale (Kobata et al. 1998, 2002; Reyes-Duarte et al. 2002; Castillo et al. 2007). The first non-pungent capsaicin analogue designed, olvanil, aimed to unveil the mechanism underlying TRPV1 activation (Dray et al. 1990; Szallasi and Blumberg 1999). However, further investigations revealed that olvanil exhibits greater apoptotic effect compared to capsaicin in C6 rat glioma cells (Jacobsson et al. 2001) and superior anti-invasive activity on human small cell lung cancer cells, with a concentration 20 times lower than that required for capsaicin (Hurley et al. 2017). Surprisingly, capsazepine, a potent capsaicin antagonist, did not block the cytotoxicity effect of olvanil when combined, suggesting a similar mechanism of action for olvanil as seen in capsaicin, where vanilloid receptors are not involved in the apoptosis of tumoral cells (Jacobsson et al. 2001).

To increase the biological activity of capsaicin analogues, the incorporation of unsaturated long chain fatty acids to the side chain is preferred (Hurley et al. 2017; Friedman et al. 2018). One group of fatty acids with attractive physiological properties are trienoic fatty acids (with three alternating double bonds) and omega-3 polyunsaturated fatty acids (n-3 PUFAs) (Aruna et al. 2016; Saini and Keum 2018). Research has shown that trienoic fatty acids are more potent than other dienoic fatty acids (with two alternating double bonds, also known as conjugated linoleic acid) (Aruna et al. 2016), and n − 3 PUFAs (*e.g.* docosahexaenoic acid) protect against chronic and metabolic diseases such as diabetes, obesity, inflammation, and osteoporosis (Saini and Keum 2018). Some trienoic fatty acids, such as α-eleostearic, jacarid acid, catalpic acid, and punicic acid, among others, are naturally found in plant seed oils (Yuan et al. 2014). Punicic acid (PA), also called trichosanic acid, is an omega 5 long chain polyunsaturated fatty acid (18:3, n-5) which is accountable for the 70–80% of fatty acid content in pomegranate seed oil (*Punica granatum*) (Alfekaik and AL-Hilfi 2016). PA shows anti-cancer, anti-inflammatory, anti-obesity, anti-diabetic, and hypolipidemic properties (Yuan et al. 2014; Alfekaik and AL-Hilfi 2016; Aruna et al. 2016). Due to these properties, PA is a promising fatty acid for the synthesis of bioactive capsaicin analogues.

Capsaicin analogues can be chemical or enzymatically synthetized. The latter technique exhibits additional advantages as enzymatic processes are usually performed in aqueous, environmental-friendly, and mild conditions (physiological pH, room temperature, and atmospheric pressure), with high specificities and selectivities (Andualema and Gessesse 2012; Sheldon and Van Pelt 2013). Additionally, enzymes can be immobilized ensuring a reusable, stable, and cost-efficient process. Although the available literature concerning enzyme immobilization is vast, the existing methodologies can be defined as carrier-bound or carrier-free (Cao et al. 2003). Among the carrier-free immobilization methods, crosslinked enzyme aggregates (CLEAs) is a simple and effective technique for the immobilization of many industrially relevant enzymes (Sheldon 2011a). Lipases, such as Lipase PS from *Burkholderia cepacia*, lipase B from *Candida antarctica* (CALB), and lipase from *Pseudomonas cepacia* are the preferred biocatalysts to obtain capsaicin analogues (Kobata et al. 2002; Reyes-Duarte et al. 2002; Liu 2009). In a recent work developed by our research group, CLEAs of CALB were employed to efficiently synthetize olvanil, a capsaicin derivative of oleic acid (Diaz-Vidal et al. 2019).

In the present work, the synthesis of capsaicin analogues from linoleic acid, docosahexaenoic acid, and punicic acid was evaluated by a chemo-enzymatic process catalyzed by CLEAs from recombinant CALB, and the catalytic efficiency was compared to commercial immobilized CALB. The cytotoxicity of the capsaicin analogues obtained, in addition to olvanil and capsaicin, was studied on human glioblastoma cells.

## Materials and methods

### Chemicals and reagents

*C. antarctica* lipase B immobilized on Immobed 150 (CALB-150, ≥ 2000 U g^−1^), *Candida rugosa* lipase (lyophilized powder, ≥ 40,000 U mg^−1^), oleic acid (technical grade, 90%), linoleic acid (≥ 95%), docosahexaenoic acid (DHA, ≥ 98%), capsaicin natural (65% capsaicin, 35% dihydrocapsaicin), capsaicin ≥ 95% (from *Capsicum* sp.), temozolomide (≥ 98% HPLC), isooctane, 2-methyl-2-butanol (2M2B), silica TLC plates (20 cm × 20 cm, fluorescent indicator), N,N-diisopropylethylamine (DIPEA), syringe filters (Supelco, Iso-Disc syringe tip filter, PTFE membrane, 0.2 μm pore size, 4 mm diameter) and Vanillylamine hydrochloride were purchased from Sigma-Aldrich (Mexico). Flash chromatography column was from ChemGlass Life Sciences (Vineland, NJ). Dulbecco's Modified Eagle Medium (DMEM), fetal bovine serum, penicillin, streptomycin, and amphotericin B were from Gibco.

Punicic acid was extracted from pomegranate seeds (*Punica granatum*) purchased at a local store (Mexico). Zeocin™ was obtained from Invitrogen (Carlsbad, CA). The restriction enzymes (DNA polymerase, and DNA ligase) and their buffers were obtained from New England Biolabs (Ipswich, MA). All other reagents and solvents were from Sigma-Aldrich (Mexico).

### Production of recombinant CALB lipases in *P. pastoris*

The sequence of CALB with its native signal peptide (GenBank: Z30645.1) was codon optimized and synthetized by Genscript® (Tucson, AZ, USA) for expression in *Pichia pastoris* (GenBank: OR227685 and Supplementary Material). *Eco*RI and *Kpn*I restriction sites were added by PCR and the resulting fragment was inserted into pGAPZB vector to generate the recombinant plasmid pGAPZB/CALB. The plasmid was linearized with *Bsp*HI and transformed into *P. pastoris* SMD1168H cells (Invitrogen™, Thermo Fisher Scientific, MA, USA) by electroporation. Transformed cells were cultured on yeast extract–peptone–dextrose (YPD) plates containing Zeocin™ (100 μg mL^−1^) and incubated at 30 °C. Positive clones were transferred to YPD plates containing 1% (v/v) of emulsified tributyrin (TG (4:0)) and the best CALB-producing strain was chosen after observing a halo formation due to lipase activity.

### Protein estimation and enzymatic activity

Protein quantification was determined with Bradford reagent using BSA as standard. The enzymatic activity was evaluated in a microplate assay by monitoring *p*-nitrophenyl butyrate (*p*NPB) hydrolysis for recombinant CALB and CALB-CLEAs. One hundred μL of 10 mM *p*NPB (20 mmol L^−1^ MOPS buffer pH 7.2, 0.5 mmol L^−1^ NaTDC, 150 mmol L^−1^ NaCl, 5 mmol L^−1^ CaCl2 and 3 g L^−1^ β-cyclodextrin) were added to 20 μL of biocatalyst.

The reaction course was followed at 410 nm for 15 min at 37 °C. One enzymatic activity unit (U) corresponds to 1 μmol of *p*NP released per min in the assay conditions.

### Preparation and characterization of CLEAs

CALB-CLEAs were prepared with a mixture of glutaraldehyde (50%, v/v, in water) to a final concentration of 20–500 mmol L^−1^ and precipitating agent (*tert*-butanol, isopropanol, acetone, acetonitrile, ethanol, polyethylene glycol (PEG) 20% (v/v), and saturated ammonium sulfate) were slowly added to recombinant CALB. Then, the aggregates were shaken in a Thermomixer (Eppendorf, Hamburg, Germany) at 30 °C, 750 rpm for 30–120 min. Immediately after, the reaction was stopped with MOPS 100 mmol L^−1^ pH 7.2 at a ratio of 1:9 and the formed CLEAs were centrifuged, washed thrice with MOPS 100 mmol L^−1^, pH 7.2 and lyophilized until further use.

CLEAs were characterized in terms of retained activity according to the following calculation (Sheldon and Van Pelt 2013):

$$Retained\;activity\;(\%)=\frac{Biocatalyst\;activity}{Initial\;activity}\;\times\;100$$

### Punicic acid extraction and purification

Whole pomegranates were peeled and de-seeded to reduce waste interference in the extraction process. The seeds were pressed to remove the juice present in them and then they were subjected to a pulp removal by constant stirring in presence of NaOH, thus providing the de-pulped seeds, these seeds were then left to dry completely before further use. Dried seeds were grounded and added to a significant amount of hexane to extract the oil present in them; the organic phase was filtrated, and the hexane removed with the use of a rotary evaporator.

Once the oil was extracted, 10 mL of *C. rugosa* lipase (50 mg mL^−1^) in MOPS buffer pH 7.2 (100 mmol L^−1^) were added to 20 mL of the pomegranate oil obtained and incubated 12 h at 40 °C. To stop the reaction 100 mL of HCl 3 mol L^−1^ were added and the lipidic fraction was extracted using 150 mL of hexane containing 1% BHT (1 mg mL^−1^), a liquid/liquid extraction was performed and the organic phase was then treated with MgSO_4_ in order to dry the solution, it was centrifuged afterwards and the solvent was removed at 40 °C with the use of a rotary evaporator; sample was stored under nitrogen atmosphere at -20 °C for further purification.

Punicic acid was purified by flash chromatography on silica gel (petroleum ether: diethyl ether, 90:10, v/v). Fractions were analyzed by thin layer chromatography (TLC) using heptane: diethyl ether: acetic acid (55:45:1, v/v/v) as eluent. Fractions containing UV absorbing free fatty acids were collected and the solvent evaporated.

### Synthesis and purification of capsaicin analogues

Capsaicin analogues were synthetized according to an optimized protocol in our research group (Diaz-Vidal et al. 2019). Briefly, reactions were carried out in an IKA Magnetic Stirrer at equimolar concentrations of vanillylamine hydrochloride (VAM-HCl) and acyl donor (oleic acid, linoleic acid, DHA, and punicic acid) at 50 °C in 5 mL amber glass vials with a total reaction volume of 1 mL. Previously, VAM-HCl was incubated with DIPEA at a ratio of 1:10 to release the salt derivative. The synthesis was performed with 1 U in *p*NPB hydrolysis of CALB-CLEAs and CALB-150 in anhydrous *tert*-Amyl alcohol (2M2B).

Reaction progress was followed by thin-layer chromatography (TLC) using as mobile phase a mixture of petroleum ether/ethyl acetate (50/50, v/v), and spots were visualized by iodine vapors. Reactions were centrifuged at 7000* g* for 5 min for the total removal of immobilized enzyme and molecular sieves. Next, the reaction solvent was fully evaporated in a rotatory evaporator and the residue resuspended in a mixture of petroleum ether/ethyl acetate at variable proportions. Purifications were carried out with silica gel flash chromatography (Silica gel 60 A, 230–400 mesh particle size) using a mobile phase of petroleum ether/ethyl acetate at variable proportions.

### Spectroscopic analysis

^1^H NMR spectra was obtained using a Bruker Ascend 400 (Billerica, Massachusetts, USA). The chemical shift values were recorded in parts per million (ppm) using CDCl3 as solvent. NMR data were obtained for *N*-(4-hydroxy-3-methoxybenzyl)oleamide (olvanil), (4Z,7Z,10Z,13Z,16Z,19Z)-*N*-(4-hydroxy-3-methoxybenzyl)docosa-4,7,10,13,16,19-hexaenamide (dohevanil), (9Z,12Z)-*N*-(4-hydroxy-3-methoxybenzyl)octadeca-9,12- dienamide (livanil), and (9Z,11E,13Z)-*N*-(4-hydroxy-3-methoxybenzyl)octadeca-9,11,13- trienamide (punivanil) synthetized by enzymatic means (Table 1). The expanded ^1^H-NMR spectrum in the region of 0.7 to 7.2 ppm for all the synthetized capsaicinoids is shown in Table S1, Supplementary Information.

**Table 1:**
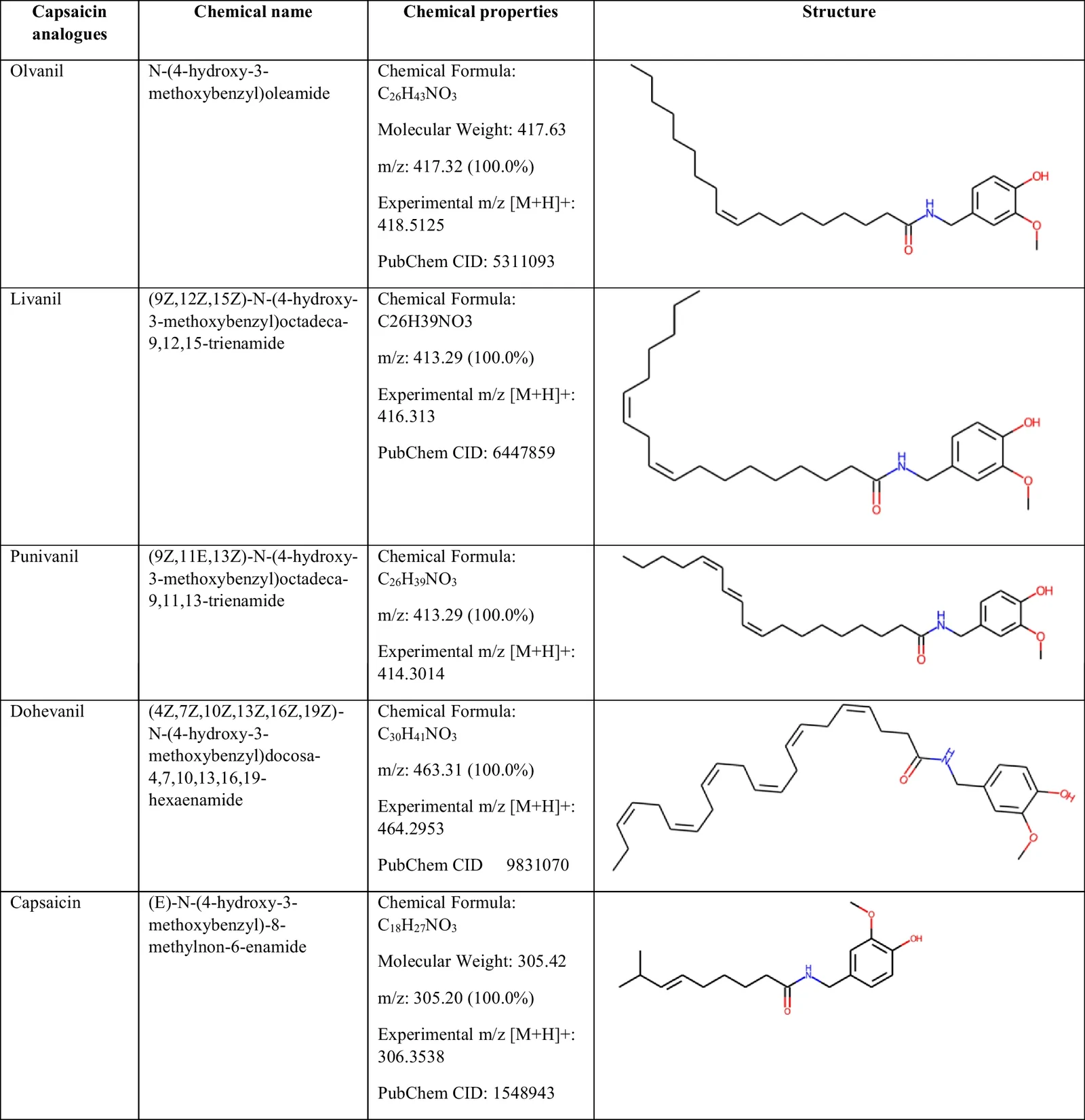
Chemical structures and properties of the enzymatically synthetized capsaicin analogues. The 2D structures were drawn with RDKit (v. 2022.03.5)

*N*-(4-hydroxy-3-methoxybenzyl) oleamide (olvanil):

^1^H NMR (600 MHz, CDCl_3_) δH 6.85 (1H, d, J = 8.0 Hz, H6), 6.80 (1H, d, J = 2.0 Hz, H2), 6.75 (1H, dd, J = 8.0, 2.0 Hz, H5), 5.62 (1H, br. s, NH), 5.58 (1H, s, OH), 5.43 – 5.23 (2H, m, H9’, H10’, + oleic acid residues), 4.35 (2H, d, J = 5.6 Hz, H7), 4.21 (m, unknown), 3.87 (3H, s, CH3O), 3.68 (m, unknown), 3.64 (3H, d, J = 0.8 Hz, unknown), 2.32 (2H, dt, J = 14.9, 7.6 Hz, H2’), 2.18 (2H, p, J = 9.1, 7.6 Hz, H8’), 2.08 – 1.95 (2H, m, H11’, + oleic acid residues), 1.76 – 1.54 (2H, m, H3’, + oleic acid residues), 1.44 – 1.16 (20H, m, H4’-H6’, H13’-H17’, + oleic acid residues), 0.87 (3H, m, H18’, + oleic acid residues).

*N*-(4-hydroxy-3-methoxybenzyl) octadeca-9,12-dienamide (livanil):

^1^H NMR (600 MHz, CDCl_3_) δH 6.85 (1H, d, J = 8.0 Hz, H6), 6.80 (1H, d, J = 2.0 Hz, H2), 6.75 (1H, dd, J = 8.0, 2.0 Hz, H5), 5.62 (1H, br. s, NH), 5.58 (1H, s, OH), 5.45 – 5.26 (4H, m, H9’, H10’, H12’, H13’, + linoleic acid residues), 4.35 (2H, d, J = 5.5 Hz, H7), 3.87 (3H, s, CH3O), 3.64 (1H, s, unknown), 2.76 (2H, t, J = 6.8, 5.7 Hz, H11’, + linoleic acid residues), 2.33 (2H, dt, J = 15.0, 7.6 Hz, H2’, + linoleic acid residues), 2.19 (2H, dt, J = 15.0, 7.6 Hz, H8’, + linoleic acid residues), 2.03 (2H, q, J = 7.2, 6.1 Hz, H14’, + linoleic acid residues), 1.63 (4H, h, J = 7.5 Hz, H3’, H15’, + linoleic acid residues), 1.51 – 1.28 (12H, m, H4’-H7’, H16’, H17’, + linoleic acid residues), 0.95 – 0.77 (3H, m, H18’, + linoleic acid residues).

*N*-(4-hydroxy-3-methoxybenzyl) docosa-4,7,10,13,16,19-hexaenamide (dohevanil):

^1^H NMR (600 MHz, CDCl_3_) δH 6.84 (1H, d, *J* = 8.0 Hz, H6), 6.78 (1H, d, *J* = 2.0 Hz, H2), 6.74 (1H, dd, *J* = 8.0, 2.0 Hz, H5), 5.73 (1H, br s, NH), 5.46 – 5.24 (12H, m, H4’, H5’, H7’, H8’, H10’, H11’, H13’, H14’, H16’, H17’, H19’, H20’, + DHA residues), 4.33 (2H, d, *J* = 5.6 Hz, H7), 3.86 (3H, s, CH3O), 3.01 – 2.70 (10H, m, H6’, H9’, H12’, H15’, H18’, + DHA residues), 2.51 – 2.32 (2H, m, H2’, + DHA residues), 2.32–2.21 (2H, m, H3’, + DHA residues), 2.06 (2H, m, H21’, + DHA residues), 1.60 (1H, s, unknown), 1.49 (1H, q, *J* = 7.5, unknown), 1.37–1.22 (m, unknown), 1.19 (s, unknown), 0.96 (3H, td, *J* = 7.5, 1.5 Hz, H22’, + DHA residues), 0.91 (t, *J* = 7.5 Hz, unknown), 0.87 (t, *J* = 7.0 Hz, unknown).

(9Z,11E,13Z)-*N*-(4-hydroxy-3-methoxybenzyl) octadeca-9,11,13-trienamide (punivanil):

^1^H NMR (600 MHz, CDCl_3_) δH 6.49–641 (2H, m, H11’, H12’), 6.04 (2H, tt, *J* = 10.7, 1.8, H10’, H13’), 5.43 (2H, dq, *J* = 10.7, 8.1 Hz, H9’, H14’), 2.34 (2H, t, J = 7.5 Hz, H2’, + punicic acid residues), 2.33–2.12 (4H, m, H8’, H15’, + punicic acid residues), 2.10 – 1.96 (m, unknown), 1.66 – 1.56 (2H, m, H3’, + punicic acid residues), 1.39–1.27 (8H, m, H4’- H7’, + punicic acid residues), 1.26–1.21 (4H, m, H16’, H17’, + punicic acid residues), 1.19 (s, unknown), 0.98 – 0.79 (3H, m, H18’, + punicic acid residues).

The molecular mass of olvanil, livanil, dohevanil, and punivanil was determined by UHRQ- TOF mass spectrometer (Xevo G2-XS QTOF, Waters, Milford, Massachusetts, USA) coupled with an ESI source. The sample was resuspended in different solvent mixtures and directly infused to an ESI source. The UHR-Q-TOF instrument was operated in positive ion mode (ES +) electrospray ionization with a capillary voltage of 2.5 kV. Data of spectrum was recorded over the mass/charge (m/z) range of 50–1200 Da and analyzed with Waters Masslynx™ Mass Spectrometry software. The molecular masses obtained are summarized in Table S2, Supplementary Information, and the obtained ion chromatographs are shown in Fig. S1, Supplementary Information.

### Scanning electron microscopy images of CLEAs

Scanning electron micrographs of CLEAs were recorded using a Mira3 LMU field emission scanning electronic microscope (FE-SEM, TESCAN, Czech Republic). Prior, the samples were freeze-dried and coated with sputtered gold.

### Cell culture

Human glioblastoma multiforme (GBM) cells U138-MG-HBT16 (U-138), U-87 MGHTB14 (U-87) and NCTC clone 929 [L cell, L-929, derivative of Strain L] were obtained from the American Type Culture Collection (ATCC, Rockville, MD). GBM cells and L-929 were grown at 37 °C in a humidified incubator under 5% CO_2_ and cultured in DMEM supplemented with fetal bovine serum (10%, w/v), penicillin (10,000 U mL^−1^), streptomycin (10,000 μg mL^−1^), and amphotericin B (25 μg mL^−1^). The culture medium was replaced daily until attaining 80% confluence.

### Viability assay

Capsaicin, olvanil, livanil, dohevanil, and punivanil were dissolved in dimethylsulfoxide (DMSO) at a final concentration of 100 mmol L^−1^. Temozolomide was selected as positive control, and the dilution was performed as instructed by the supplier. The solutions were then sterilized with syringe filters and kept frozen until use.

U-138, U-87 and L-929 (as control) cells (5,000 cells/well in 200 μL of DMEM) were cultivated in 96-well plates for 72 h at 37 °C, under 5% CO_2_, using the 3D model based on Matrigel™, (Corning, Matrigel, Matrix basement membrane) at 50%. Next, the cells were incubated with 50, 100, 200, and 400 μmol L^−1^ of capsaicin analogues and temozolomide (300, 600, 900, and 1200 μmol L^−1^) diluted in DMEM for GBM cells and 200, 300 and 500 µmol L^−1^ for L-929 cells. The cytotoxicity of the vehicle (DMSO, 1%, v/v) in DMEM) was also tested. After 24 h of incubation, the cytotoxic effect of capsaicin, capsaicin analogues, temozolomide, and DMSO was determined using the 3-(4,5-dimethylthiazol-2-yl)-2,5-diphenyltetrazolium bromide (MTT) assay as per manufacturer’s instruction. The absorbance was measured at 570 nm in a microplate reader (Bio-Rad). Three replicates were used for each capsaicin, capsaicin analogue, temozolomide, and vehicle concentration.

### Induction of cell death by Apoptosis

#### Detection of phosphatidylserine externalization

For this determination, monolayer cultured GBM were used with a density of 200,000 cells per well in a 24-well plate in triplicate. After 24 h, the medium was removed and the studied molecules were added at the following concentrations: capsaicin at 300 μmol L^−1^, olvanil and dohevanil at 200 μmol L^−1^, and temozolomide (TMZ) at 600 μmol L^−1^. After 3 h of incubation, the molecules were removed, the cells were detached with EDTA at 2 mmol L^−1^ and worked according to the Annexin V protocol (Annexin V conjugated with Alexa-Fluor 488, Invitrogen Cat. No. 13201 PHN1010 and PHN1008). The detection of phosphatidylserine externalization was measured at 533 nm and 575 nm using a Flow Cytometer (Guava EasyCyte 5, Millipore).

#### Caspase-3 activation

3D culture of GBM cells with the Matrigel base model was performed in a culture chamber system on an 8-well slide (Thermo-Scientific, Cat. No. 154461-PK, Nunc Lab-Tek™ II Chamber Slide System), at a density of 20,000 cells per well. The cells were incubated for 72 h with culture medium at 37 °C with an air atmosphere of CO_2_ 5%. Then, the following capsaicin analogue concentrations were added: capsaicin and livanil at 300 μmol L^−1^, dohevanil at 200 μmol L^−1^. The culture was incubated for 6 h. Next, the culture medium was removed and incubated with a solution of paraformaldehyde 4% (v/v) in 0.1 mol L^−1^ phosphate buffer at pH 7.4 (PBS), for 2 h. Immunofluorescence assay for caspase 3 (Abcam, ab-13847 cysteine-protease-3) was performed with a dilution of 1:500 and allowed to incubate overnight at 4 °C. Alexa-Fluor 488 secondary antibody was bound for 2 h in the dark (1:500, Vector Laboratories), protected with a drop of mounting medium with 4',6-diamidino-2-phenylindole, di-hydrochloride (Fluoroshield, Abcam 104,139), and later analyzed under fluorescence microscopy (Leica DM4 DF7000T, Leica Biosystems, Wetzlar, Germany). The obtained images were analyzed with the Leica Application Suite software (LAS). For each taken image taken, the degree of fluorescence intensity was kept constant. The total cells (DAPI) and cells labeled with Caspase 3 (Green) were counted in 10 fields (1.5 mm2) for each well and each group (3 wells). The percentage of labeled cells was calculated using the double-blind technique.

### Statistical analysis

All data are expressed as mean ± standard deviation (SD). Statistical analysis was conducted with the GraphPad Prism software, v. 6 (GraphPad Software, San Diego, CA, USA). Differences on cell viability were analyzed by one-way and two-way analysis of variance (ANOVA) followed by Tukey post-hoc to determine whether the differences from the studies groups were significant from those of the capsaicin analogues treated groups. In all cases, the level of statistical significance was set to *p* < 0.05, 0.001, 0.0005 or 0.0001.

## Results

### Preparation and characterization of CLEAs

Prior to the preparation of CALB-CLEAs, the crude fermentation extract of *P. pastoris* containing recombinant CALB, among contaminant proteins, was concentrated 32 times and dialyzed with Milli-Q water by ultrafiltration with a 10 kDa membrane, in order to remove salts from the medium. The concentrated crude fermentation extract of CALB contained 0.8 g L^−1^ of total protein. The enzymatic activity of CALB prior to immobilization was 38 U mL^−1^.

Before the CLEAs preparation, the best precipitation conditions were initially established. For this, 1 part of CALB was incubated with 9 parts of different precipitating agents for 30 min at 30 °C. In a previous work from our group, we described the preparation and optimization of CLEAs of recombinant CALB for the synthesis of olvanil (Diaz-Vidal et al. 2019). The results indicated that CALB precipitated with isopropanol and cross-linked with 150 mmol L^−1^ for 60 min gave active and highly efficient crystal-shape immobilizates. Thus, we selected the same precipitation and cross-linking conditions for the present synthesis of capsaicin analogues.

### Scanning electron microscopy images of CLEAs

**Fig. 1:**
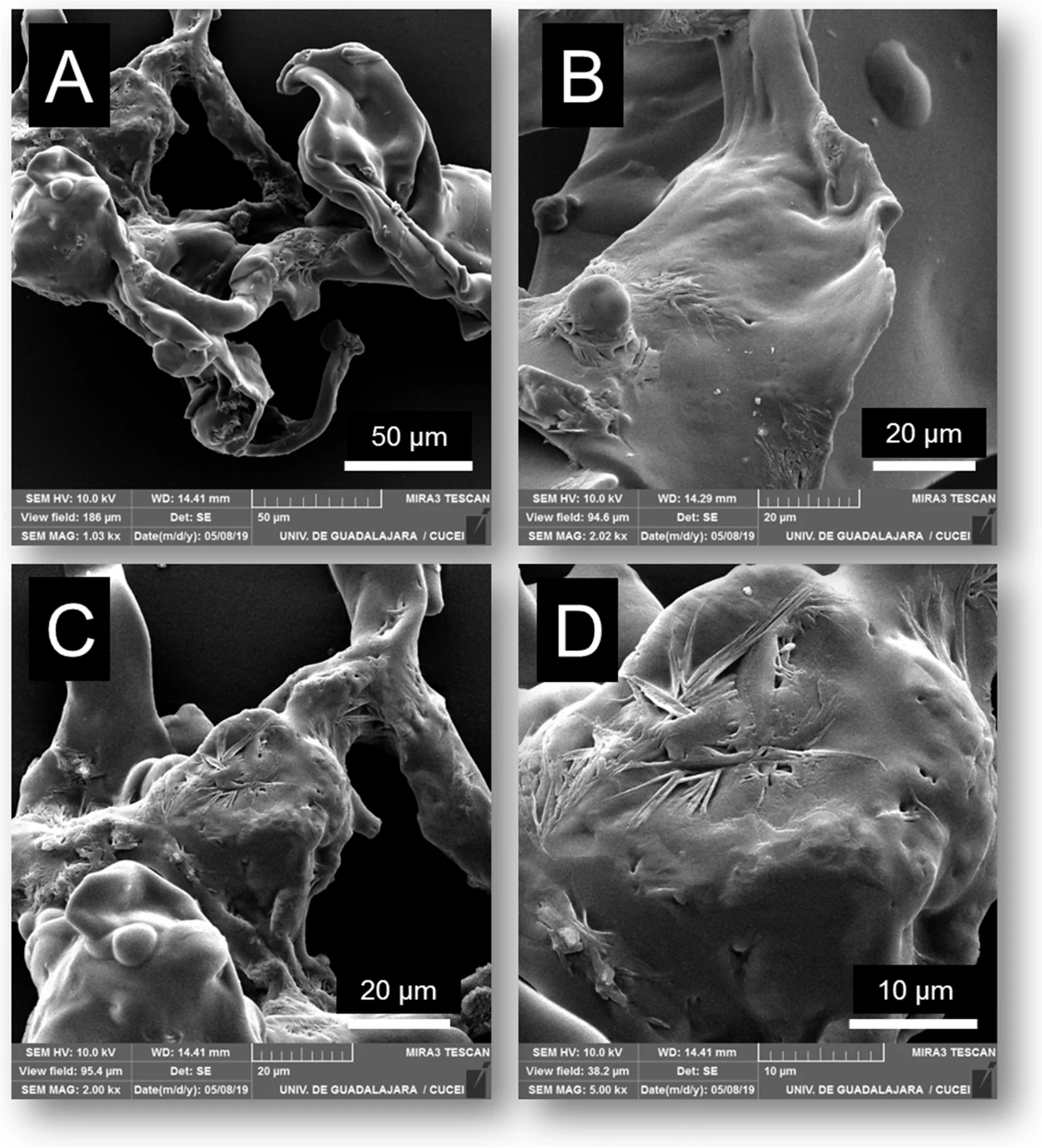
Scanning electron micrographs of the structure of CALB-CLEAs at **A)** 1,030 × magnifications, **B)** 2,200 × magnifications, **C)** 2,000 × magnifications, and **D)** 5,000 × magnifications. CLEA preparation conditions: isopropanol as precipitating agent, 150 mM glutaraldehyde for 60 min at 30 °C

The morphology of CLEAs depends on enzyme type, precipitant agent and the microenvironment of the enzyme when cross-linking (Zerva et al. 2018). SEM micrographies revealed the formation of clusters with irregular, cluster shapes and low porosity for CALB-CLEAs (Fig. 1).

### Synthesis of capsaicin analogues

The synthesis of capsaicin analogues was conducted on 2M2B at 50 °C with equimolar concentrations of VAM-HCl and oleic acid, linoleic acid, DHA, and punicic acid as acyl donors. The amount of biocatalyst was equivalent to 1 U on the hydrolysis of *p*NPB (20 mg and 38 mg of CALB-CLEAs and CALB-150, respectively). Table 2 shows the concentration and conversion values of livanil, dohevanil and punivanil synthesis using CALB-150 at 50 °C for 24, 48 and 72 h. The highest conversion was achieved when punicic acid was used as acyl donor. At 48 h of reaction, 19.4 g L^−1^ of punivanil were synthetized with 73% of conversion. Surprisingly, at 72 h, the conversion of punivanil dramatically decreased 3.3-fold. In contrast, the synthesis of livanil increased steadily through time, achieving the maximum conversion yield at 72 h (58.3% conversion to livanil). Dohevanil was obtained in moderate amounts, and similar conversion values were obtained at 48 and 72 h.

**Table 2:** Concentration and conversion values of livanil, dohevanil, and punivanil at 24, 48, and 72 h catalyzed by CALB immobilized on immobead 150

	24 h		48 h		72 h	
Compound	Concentration (g L^−1^)	Conversion (%)	Concentration (g L^−1^)	Conversion (%)	Concentration (g L^−1^)	Conversion (%)
Livanil	4.98 ± 0.37	24	8.66 ± 0.33	41.7	12.1 ± 0.67	58.3
Dohevanil	4.2 ± 0.6	18.1	5.37 ± 1.08	23.2	5.62 ± 1.46	24.2
Punivanil	17 ± 4.28	66.6	19.42 ± 0.55	73	5.85 ± 0.45	22

The concentration and conversion values obtained in the synthesis of livanil, dohevanil, and punivanil catalyzed by recombinant CALB-CLEAs is resumed in Table 3. Overall, the catalytic performance displayed by CALB-CLEAs was low in comparison to commercial CALB-150. In contrast to CALB-150, CALB-CLEAs displayed a strong preference for linoleic acid as acyl donor. At 72 h, the conversion to livanil was 69.7%, the highest conversion value obtained in comparison to the rest of acyl donors. In contrast, DHA gave the poorest catalytic results, and only 8.3% of conversion to dohevanil was achieved at 72 h. Similar to CALB-150, the synthesis of punivanil reached a maximum at 48 h, followed by a decrease in conversion, where presumably the hydrolysis of the product predominated. The ^1^H NMR spectra of the isolated capsaicinoids showed typical chemical shifts of the vanillyl ring, 3-OCH_3_, 4-OH, NH group, and long-chain acyl moieties for olvanil, livanil, and dohevanil (Kobata et al. 2010; Roby et al. 2015). For punivanil, the chemical signals of 3-OCH_3_, double bonds, and C-bonds of the aliphatic chain were easily detected; however, we were unable to completely identify the H and NH group peaks of the vanillyl ring. This could be explained by an aromatic solvent induced shift (Cao et al. 2007). The enzymatically synthetized capsaicin analogues were also subjected to mass spectrometry analysis. The theoretical molecular masses were in agreement with the experimentally measured m/z values (Supplementary Table S2 and Fig. S1).

**Table 3:** Concentration and conversion values of livanil, dohevanil, and punivanil at 24, 48 and 72 h catalyzed by CALB-CLEAs. The CALB-CLEAs were obtained with isopropanol as precipitating agent and cross-linked with 150 mmol L^−1^ of glutaraldehyde for 60 min

	24 h		48 h		72 h	
Compound	Concentration (g L^−1^)	Conversion (%)	Concentration (g L^−1^)	Conversion (%)	Concentration (g L^−1^)	Conversion (%)
Livanil	1.83 ± 0.11	8.8	10.1 ± 1.13	48.6	14.47 ± 0.40	69.7
Dohevanil	0.30 ± 0.13	1.3	1.26 ± 0.12	5.4	1.91 ± 0.27	8.3
Punivanil	7.18 ± 0.8	24.4	12.09 ± 1.77	41.2	8.88 ± 5.29	30.3

### Effect of capsaicin and capsaicin analogues on the cytotoxicity of glioblastoma cells

**Fig. 2:**
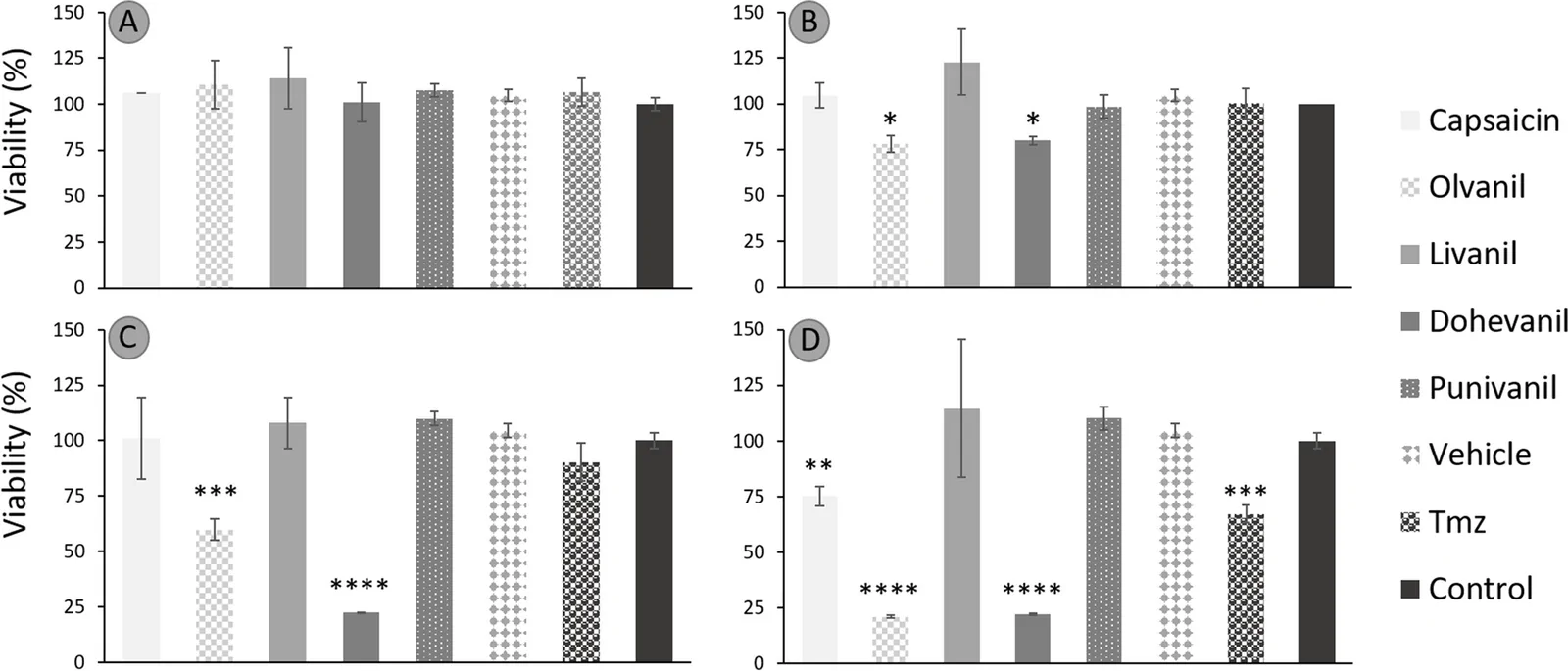
Average viability percentage of U-138 cell line in a concentration-dependent manner: **A**) 50 μmol L^−1^, **B**) 100 μmol L^−1^, **C**) 200 μmol L^−1^, and **D**) 400 μmol L.^−1^. Data are expressed as the average viability percentage ± standard deviation at different doses of capsaicin, capsaicin analogues, control (culture medium), temozolomide (TMZ, as positive control) and vehicle (DMSO) and analyzed using one-way ANOVA test followed by Tukey post-hoc. Differences were considered significant when *p < 0.05, **p < 0.001, ***p < 0.0005 or **** *p* < 0.0001

**Fig. 3:**
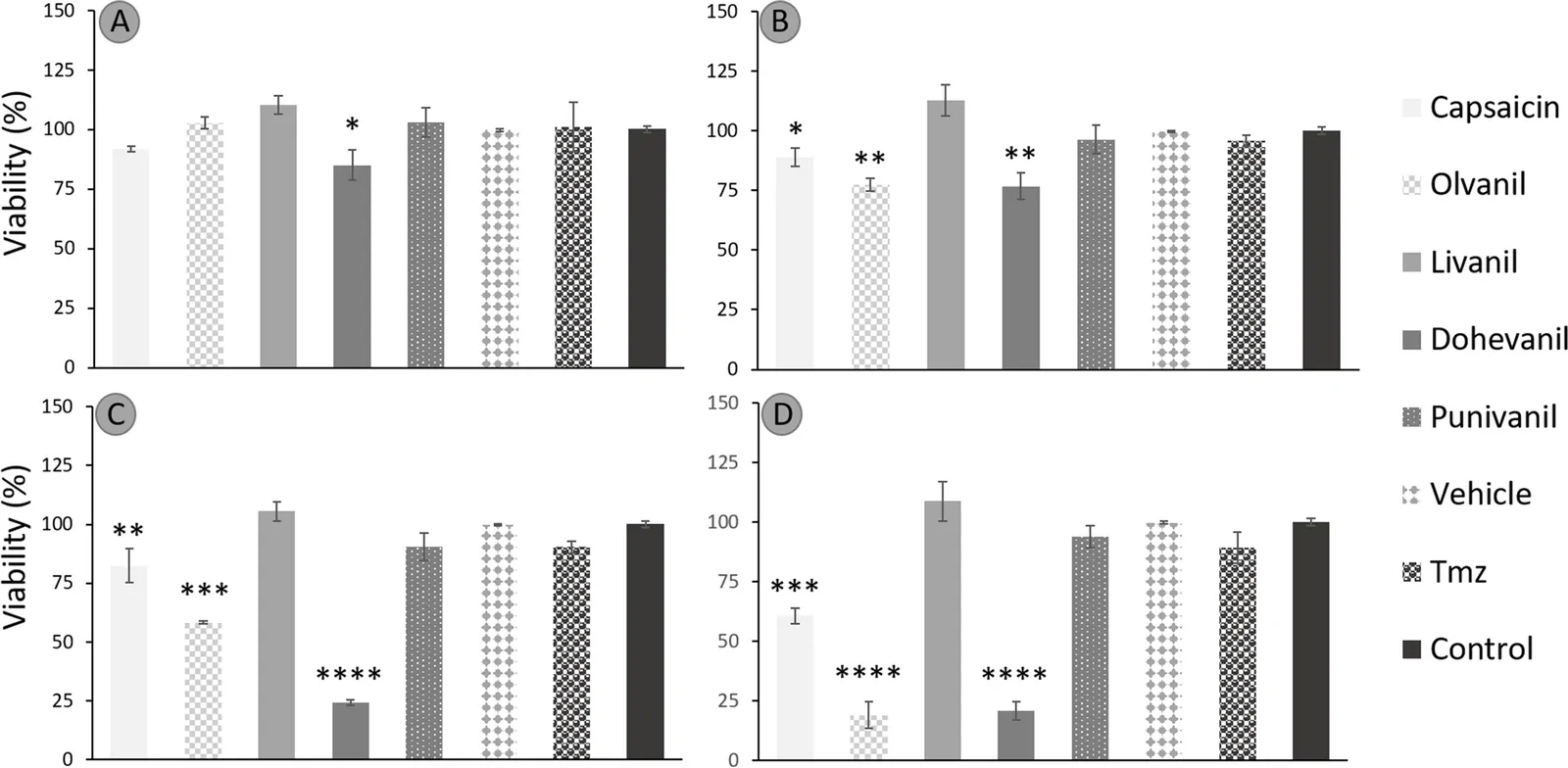
Average viability percentage of U-87 cell line in a concentration-dependent manner: **A**) 50 μmol L^−1^, **B**) 100 μmol L^−1^, **C**) 200 μmol L^−1^, and **D**) 400 μmol L.^−1^. Data are expressed as the average viability percentage ± standard deviation at different doses of capsaicin, capsaicin analogues, control (culture medium), temozolomide (TMZ, as positive control) and vehicle (DMSO) and analyzed using one-way ANOVA test followed by Tukey post-hoc. Differences were considered significant when *p < 0.05, **p < 0.001, ***p < 0.0005 or **** *p* < 0.0001

The viability of U-138 and U-87 glioblastoma cells treated with increasing concentrations of capsaicin, capsaicin analogues (olvanil, livanil, dohevanil, and punivanil), and temozolomide for 24 h was determined by the MTT assay. Capsaicin analogue type and concentration had a statistical effect on cell viability. Increasing concentrations of capsaicin, olvanil, and dohevanil had a significant effect on the viability of U-138 and U-87 cells, whereas livanil and punivanil did not show a significant cytotoxic effect (Fig. 2 and 3). Photomicrographs of viable and non-viable cell lines due to capsaicinoid exposition are shown in Supplementary Fig. S2.

U-138 cells treated with capsaicin maintained their viability at capsaicin concentrations ≤ 100 μmol L^−1^. However, at capsaicin concentrations ≥ 400 μmol L^−1^, the viability was reduced to 65 ± 3.6%. In contrast, U-87 cells required less capsaicin concentration to induce a cytotoxic effect. The viability was reduced to 91.7% at the lowest capsaicin concentration assessed (50 μmol L^−1^), and the maximum cytotoxic effect was observed with 400 μmol L^−1^ of capsaicin with a viability of 60.66 ± 2.7%. The viability of U-138 cells treated with olvanil at concentrations ≥ 100 μmol L^−1^ had a significant effect in cytoxicity, recording a minimum of 21.1 ± 0.4% in viability at the highest olvanil concentration evaluated (400 μmol L^−1^). U-87 cells treated with olvanil responded similar to U-138 cells. Concentrations ≥ 200 μmol L^−1^ significantly reduced the cell viability from 77.3 ± 2.2% with 100 μmol L^−1^ olvanil to 18.8 ± 4.6% with 400 μmol L^−1^. The cytotoxic effect of olvanil was more notorious for U-87 cells, as also observed for capsaicin.

U-138 cell viability was diminished to 79.9 ± 1.7% when exposed to dohevanil concentration of 100 μmol L^−1^. However, at a concentration of 200 μmol L^−1^, the cell viability drastically decreased to 22.3 ± 0.2%. Increasing the concentration of dohevanil at 400 μmol L^−1^ had no effect on the cytotoxicity of U-138 cells. For U-87 cells, the cytotoxic effect of dohevanil was more notorious at lower concentrations (50 and 100 μmol L^−1^) with a cell viability of 85.1 ± 5.1% and 76.8 ± 4.5%, respectively. The cell viability effect at concentrations ≥ 200 μmol L^−1^ was similar to that observed for U-138 cells (24.1 ± 0.9 20.6 ± 3.1 for 200 and 400 μmol L^−1^, respectively).

 Conversely, the studied concentrations of livanil and punivanil (50–400 μmol L^−1^) had no significant effect on the cytotoxicity of U-87 and U-138 cells.

The induction of cell death by apoptosis in GBM cells due to the effect of capsaicin and its analogues was determined by the detection of phosphatidylserine externalization and the expression of caspase-3. Capsaicin analogues tested after 3 h promoted the binding of annexin V to externalized phosphatidylserine with a positive percentage of cells of 60.6 ± 7.2, 65.5 ± 5.7 and 67.5 ± 3.9% for capsaicin (300 μmol L^−1^), olvanil and dohevanil (200 μmol L^−1^), respectively. The positive control temozolomide (600 μmol L^−1^) promoted a value of 40.8 ± 7.2% (Supplementary Fig. S3).

The expression of active caspase-3 in GBM cells could be observed by fluorescense photomicrographs (Supplementary Fig. S4). For cells without the caspase-3 labeling marker using Alexa-Fluor 488 (green), only the nucleus was stained with DAPI (blue). After 6 h of exposure, the positivity for capsaicin and dohevanil was 19.1 ± 2.6% and 73.5 ± 7.6%, respectively, with a difference to the control group of 5.6 ± 1.6. As expected, livanil did not promote the expression of caspase-3, showing a positivity percentage of 3.5 ± 1.5% with no significant differences compared to the control group.

**Fig. 4:**
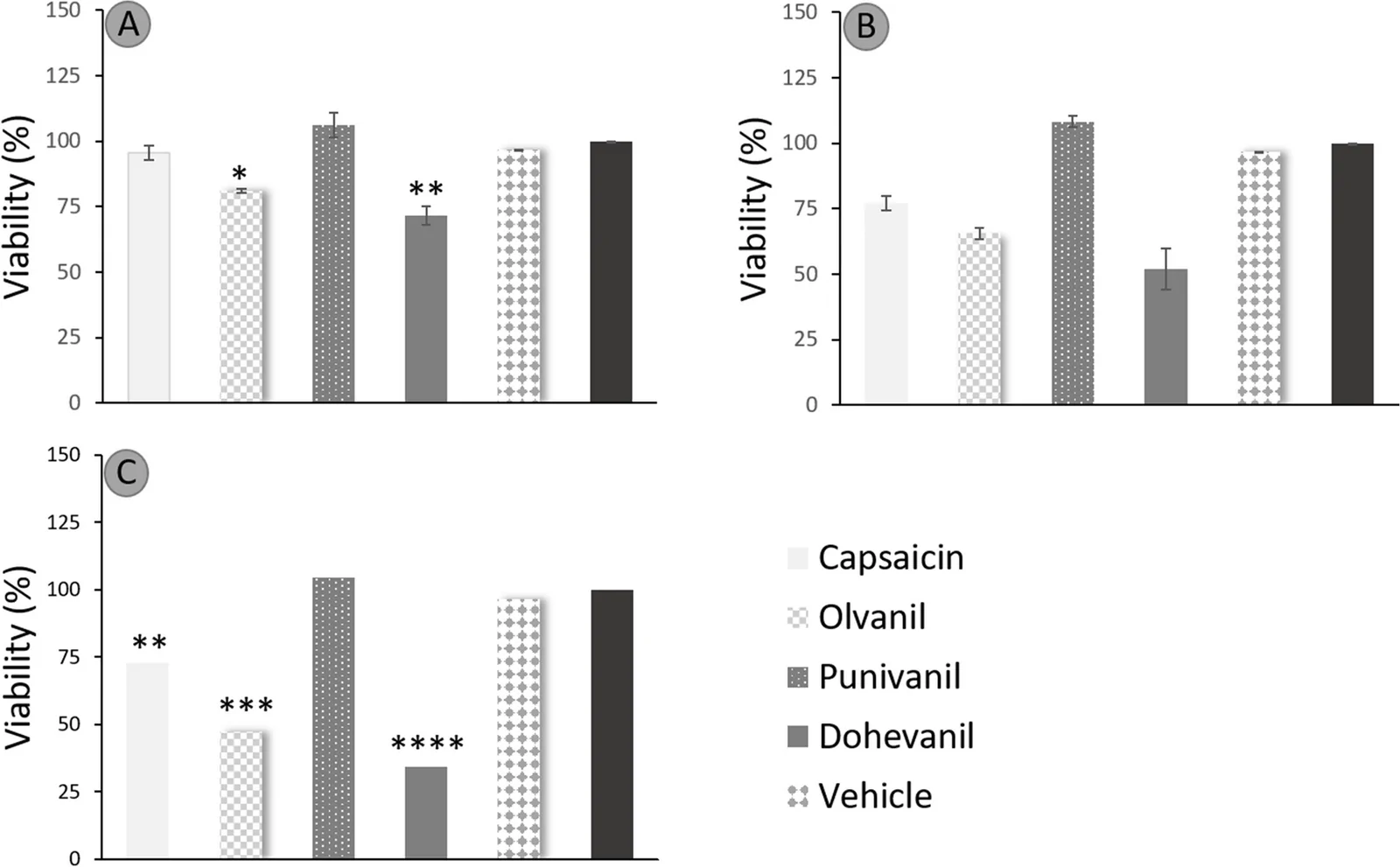
Average viability percentage of L929 cell line in a concentration-dependent manner: **A**) 200 µmol L^−1^, **B**) 300 µmol L^−1^ and **C**) 500 µmol L.^−1^. Data is expressed as the average viability percentage ± standard deviation at different doses of capsaicin, capsaicin analogues, control (culture medium), temozolomide (TMZ, as positive control) and vehicle (DMSO) and analyzed using one-way ANOVA test followed by Tukey post-hoc. Differences were considered significant when *p < 0.05, **p < 0.001, ***p < 0.0005 or **** *p* < 0.0001

The cytotoxicity test in the L-929 line presented a lower level of cytotoxicity compared to GBM cells (Fig. 4). The utilization of dohevanil at a concentration of 500 µmol L^−1^ in L-929 cells results in an average cell viability of 34%. Meanwhile, at a concentration of 400 µmol L^−1^, the average viability was 22% and 24% in U-138 and U-87 cells, respectively.

Likewise, cells incubated with olvanil showed a viability of 47% at a dose of 500 µmol L^−1^, whereas in lines U-138 and U-87, the viability at 400 µmol L^−1^ was 21% and 18%, respectively. Punivanil did not show any type of cytotoxicity.

## Discussion

Capsaicin analogues from punicic acid, linoleic acid, and DHA were obtained by a chemoenzymatic process catalyzed by commercial CALB-150 and CALB-CLEAs. The capability of CALB to efficiently synthetize and hydrolyze amides has been previously described (Anderson et al. 1998; Torres-Gavilan et al. 2006). In particular, commercial CALB (Novozym 435) has mediated the synthesis of olvanil, rinvanil, and *N*-vanillyltetradecanamide with efficacies greater than 70% (Kobata et al. 1999; Reyes-Duarte et al. 2002; Castillo et al. 2008).

The catalytic efficiency of commercial CALB-150 was compared to recombinant CALB-CLEAs. The CLEAs were obtained directly from the fermentation extract of a deficient protein strain of *P. pastoris*. In a previous work, we observed that recombinant CALB precipitated with isopropanol and further cross-linked with 150 mmol L^−1^ of glutaraldehyde for 60 min led to active, robust, and efficient CLEAs in the synthesis of olvanil (Diaz-Vidal et al. 2019). In the present work, the same immobilization conditions were selected for the synthesis of long-chain *N*-vanillylamides.

Overall, the performance of commercial immobilized CALB on immobead 150 was greater than that of recombinant CALB-CLEAs. This might indicate that the CLEA preparation conditions selected for the synthesis of these particular long-chain capsaicin analogues were not optimum. Immobilization protocols can greatly affect the selectivity of a certain enzyme in relation to changes in the tridimensional structure of the enzyme (Rodrigues et al. 2013). Hydrophobic supports tend to improve the catalytic performance of lipases due to interfacial activation (Fernandez-Lafuente et al. 1998; Rodrigues et al. 2013). This could explain the poor catalytic performance obtained with CALB-CLEAs in contrast to commercial CALB-150. Although the CLEA methodology is a carrier-free method, different approaches can be aimed to cause an interfacial activation on an enzyme without the need of hydrophobic supports. In the presence of an imprinting template, such as polymers, substrates, surfactants, detergents, etc., an open conformation can be mimicked and further locked by the addition of a cross-linker (Mingarro et al. 1995; Abahazi et al. 2014; Zhang et al. 2017). This technique is known as molecular bioimprinting, and besides its simplicity and low cost, it can modify the specificity, stability, and selectivity of lipolytic enzymes (Fishman and Cogan 2003; Foresti et al. 2005).

As many parameters influence the catalytic performance of CLEAs, it is important to optimize its preparation by screening a variety of precipitant agents, cross-linking agent type and concentration, agitation rate, additives, etc. (Sheldon 2011b). In this work, we selected the optimum CLEA preparation conditions for the synthesis of olvanil, but as observed, these conditions are unfavorable for the synthesis of other *N*-vanillylamides.

Therefore, in order to increase the yield of livanil, dohevanil, and punivanil synthesis, additional CLEA optimization procedures should be performed for each reaction. Although the synthesis of livanil and dohevanil has been previously described by chemical means (Melck et al. 1999; Jin et al. 2002; Sumithran et al. 2012), this is the first report concerning the lipase mediated synthesis of these compounds. However, as far as we are concerned, our report is the first and only concerning the synthesis of punivanil, a capsaicin analogue from punicic acid.

According to the morphology of the CALB-CLEAs obtained in this work, the immobilizates can be classified as *“type 2”*, which are non-defined clusters (Schoevaart et al. 2004). In general, the structures had a highly irregular, clustered shape, which is a typical feature of highly glycosylated, hydrophilic enzymes. However, at some magnifications, some “*ball type or type 1”* structures could be observed among the flat surface (Schoevaart et al. 2004). The formation of *“type 1”* structures is caused when the enzyme is highly lipophilic, as in the case of CALB. As the CLEAs in this work were immobilized directly from the fermentation broth of *P. pastoris*, the presence of contaminant proteins of unknown hydrophobicity and lipophilicity is expected. This mixture can help explain why CALB-CLEAs are a combination of both *“type 1”* and *“type 2”* structures. However, based on our results, the CLEA preparation conditions selected for our CALB-CLEAs generated immobilizates with poor catalytic performance in the synthesis of capsaicin analogues.

As expected, capsaicin analogues and temozolomide demonstrated a dose-response cytotoxic effect for U-87 and U-138 glioblastoma cell lines. Although the molecular mechanisms underlying the cytotoxicity on U-138 cells has not been elucidated, similar reports with U-87 cells pointed out a TRPV1-independent mechanism in where the apoptosis pathway is activated. Jeon and colleagues found that cell viability of U-87 cells incubated with capsaicin decreased in a dose-dependent manner (Jeon et al. 2012). Capsaicin analogues, especially dohevanil and olvanil, were able to promote the extracellular expression of phosphatidylserine in MG U-138 cells, as well as caspase 3, which serve as indicators that these compounds could be acting in apoptotic pathways as observed in prior studies. Morphological changes, down-regulation of BCL-2 expression, up-regulation of Bax expression, and DNA fragmentation proved that apoptosis occurred during capsaicin induced cytotoxicity of U-87 cells. Thus, apoptosis occurred via activation of the p-38 MAPK signaling pathway and the mitochondrial pathway of BCL-2/Bax. In contrast to what has been reported for other cell lines, caspases did not participate in the capsaicin-induced apoptosis of U-87 cells (Jeon et al. 2012). However, the reported cell viability values were slightly lower than the ones obtained in this work. Values of ~ 60% and ~ 50% cell viability were determined after incubation with 200 and 400 μmol L^−1^ of capsaicin, respectively.

A similar loss of cell viability (~ 80%) was also observed for LN-18 glioblastoma cells after 24 h of incubation with 200 μmol L^−1^ capsaicin (Szoka and Palka 2020). However, the effect of 400 μmol L^−1^ capsaicin after 24 h of incubation was more detrimental on cell viability, and values around ~ 30% were determined. Similarly, capsaicin activated the intrinsic apoptosis pathway of LN-18 cells, which is regulated by Bcl-2 proteins, and increased levels of PPARɣ expression. Indeed, a combination therapy of capsaicin analogues with PPARɣ agonists, such as thiazolidinediones, is a promising strategy for the treatment of several cancer cell lines (Hurley et al. 2017; Szoka and Palka 2020).

Dohevanil, a capsaicin analogue from DHA, was the most cytotoxic capsaicin analogue on both U-138 and U-87 cells, tested both in cytotoxicity tests with MTT, as well as apoptotic pathways, expressed by the increase in phosphatidylserine translocation and the expression of caspase 3, as shown in other research with other types of cancers. Dohevanil required less dose to achieve a significant cytotoxic effect. The cytotoxic effect of dohevanil has also been studied for MCF-7 human breast cancer cells. Similar to our results, Tuoya et al*.* observed that the apoptosis induction caused by dohevanil was more potent than that of capsaicin (Tuoya et al. 2006). The cytotoxic effect of olvanil was also significant on both U-138 and U-87 cells and the data suggests that the potency of olvanil might be greater than that of capsaicin. Similar results have also been seen with human small cell lung cancer cells (Hurley et al. 2017) and C6 rat glioma cells and EFM-19 breast cancer cells (Melck et al. 1999).

Livanil and punivanil did not show a significant cytotoxic effect on U-87 and U-138 glioblastoma cells. However, both capsaicin analogues increased cell viability by ~ 9–30%. The prospective application of these capsaicin analogues is still unexploited, but according to our results, these compounds can be envisaged as cell growth promoters in cases where cell protection is required.

L-929 cells are mouse connective tissue cells, with fibroblast morphology from the ATCC. As these cells do not present any pathology, they are commonly used to carry out toxicity studies. Nevertheless, these capsaicin analogues were employed for testing, revealing cytotoxic properties. Yet, upon analyzing specific doses and comparing these cells with tumor cells, they exhibited a lower level of cytotoxicity.

If capsaicin analogues prove effective as antitumor therapies, their utilization alongside specific adjuncts could enable targeted action towards tumors without affecting healthy cells. Nevertheless, at lower doses, their cytotoxicity in healthy cells is comparatively lower than in tumor cells.

## Supplementary Information

Below is the link to the electronic supplementary material.

Supplementary file1 (PDF 1.19 MB)

## Data Availability

The data that support the findings of this study are available from the corresponding author, JAR, upon reasonable request.
