# Supplementary material for: Long chain capsaicin analogues synthetized by CALB-CLEAs show cytotoxicity on glioblastoma cell lines
Source: Appl Microbiol Biotechnol. 2024 Jan 12;108(1):106. doi: 10.1007/s00253-023-12856-y (PMC10786984; doi:10.1007/s00253-023-12856-y)
Supplement: Supplementary file 1 — Supplementary file1 (PDF 1.19 MB) [file 253_2023_12856_MOESM1_ESM.pdf]

Applied Microbiology and Biotechnology

Long chain capsaicin analogues synthetized by CALB-CLEAs show cytotoxicity on glioblastoma cell lines

Tania Diaz-Vidal[1], Vicente Paúl Armenta-Pérez[1], Luis Carlos Rosales-Rivera[2], Georgina Cristina Basulto-Padilla[1], Raúl Balam Martínez-Pérez[1a], Juan Carlos Mateos-Díaz[1], Yanet K. Gutiérrez-Mercado[3b], Alejandro A. Canales-Aguirre[3], Jorge A. Rodríguez[1*]

[1] *Biotecnología Industrial, Centro de Investigación y Asistencia en Tecnología y Diseño del Estado de Jalisco, CIATEJ, 45019 Zapopan, Mexico*

[2] *Departamento de Ingeniería Química, CUCEI, Universidad de Guadalajara, 44430 Guadalajara, Mexico*

[3] *Unidad de Evaluación Preclínica, Unidad de Biotecnología Médica y Farmacéutica, Centro de Investigación y Asistencia en Tecnología y Diseño del Estado de Jalisco, CIATEJ, 44270 Guadalajara, Mexico*

*Corresponding author: J.A. Rodríguez; email: jrodriguez@ciatej.mx; phone +52 33 33 45 52 00 ext. 2108; fax +52 33 33 45 52 00 ext. 1001

[a] Present address: *Departamento de Biotecnología y Ciencias Alimentarias, Instituto Tecnológico de Sonora, 85137 Ciudad Obregón, Mexico*

[b] Present address: *Laboratorio Biotecnológico de Investigación y Diagnóstico, departamento de Clínicas, División de Ciencias Biomédicas, Centro Universitario de los Altos, Universidad de Guadalajara, Tepatitlán de Morelos, Jalisco, Mexico*

Supplementary Material

Table S1. Expanded ^{1}H-NMR spectrum in the region of 0.7 to 7.2 ppm for the enzymatic synthesis of olvanil, livanil, dohevanil, and punivanil (from left to right, up to down),

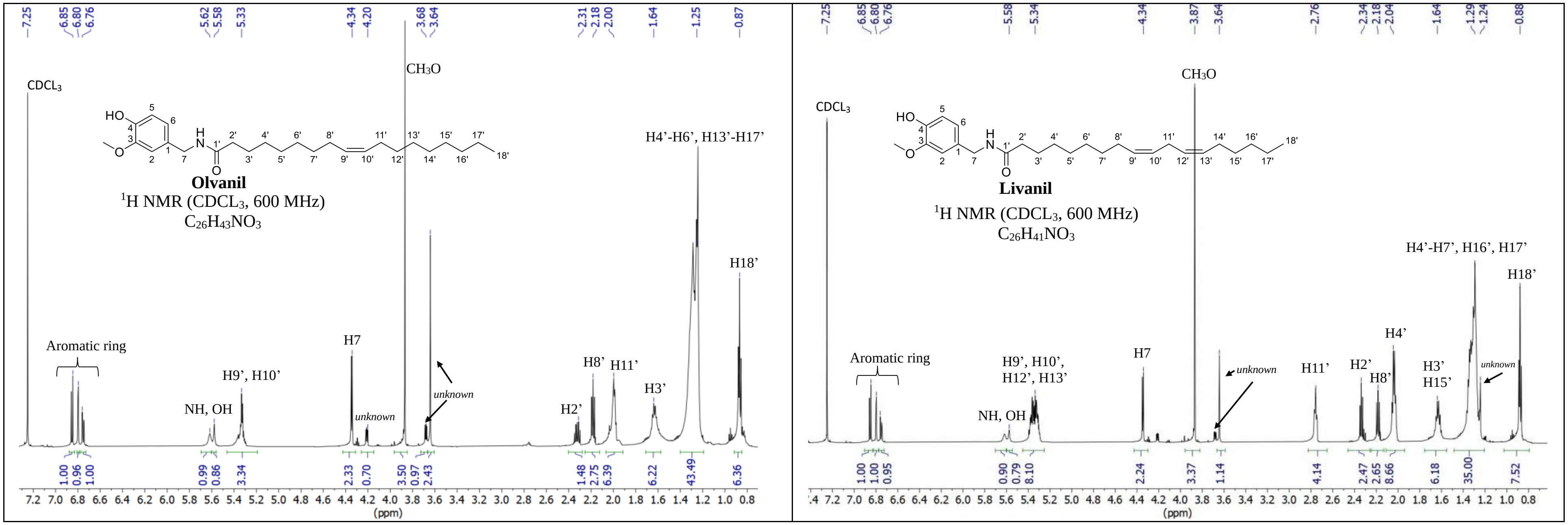

Table S1 continued

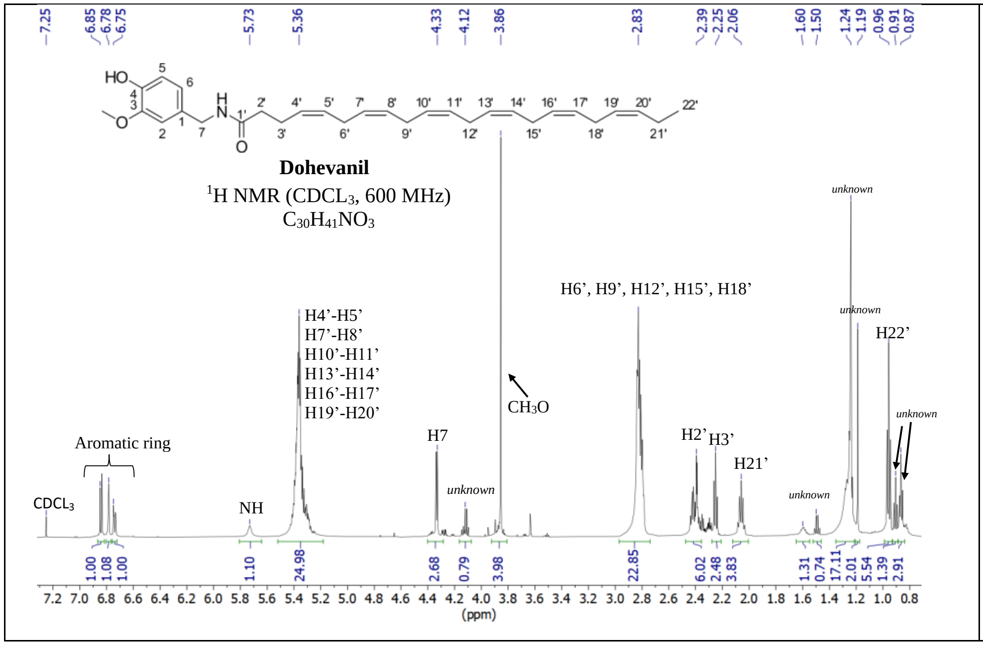

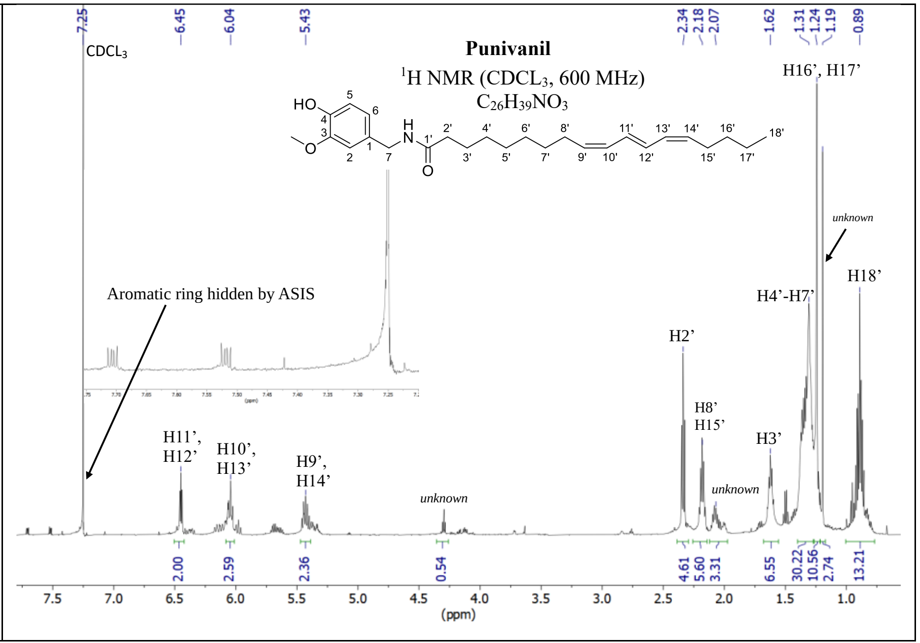

Table S2. MS parameters of N-CAPS obtained with CALB-CLEAs by using LC–MS–MS in the positive ESI mode. Collision energy 6.0 kV.

**Compound**	**Solvent system**	**Precursor ion (theoretical m/z)**	**Theoretical relative intensity**	**Product ions (experimental m/z)**	**Relative intensity to base peak**
Livanil	Methanol/water (7/3)+formic acid 0.5%	415.3086	100	416.313	100
		416.312	28.1	417.3074	25.9
		417.3154	2.7	418.3239	4.8
		417.3154	1.1	418.3281	3.9
Punivanil	Acetonitrile/water (7/3)+phosphoric acid 0.1%	413.293	100	414.3014	100
		414.2963	28.1	415.1266	28.9
		415.2997	2.7	415.306	26.6
		415.2997	1.1	416.1281	2.6
Dohevanil	Methanol/water (7/3)+formic acid 0.5%	463.3086	100	464.2953	100
		464.312	32.4	465.3012	32.7
		465.3154	5.1	466.2994	5.4
Olvanil	Methanol/formic acid (100/1).	417.32	100	418.5125	100
		418.32	28.1	419.5137	63.6
		419.33	2.7	420.5204	12.3
		419.33	1.1	-	-

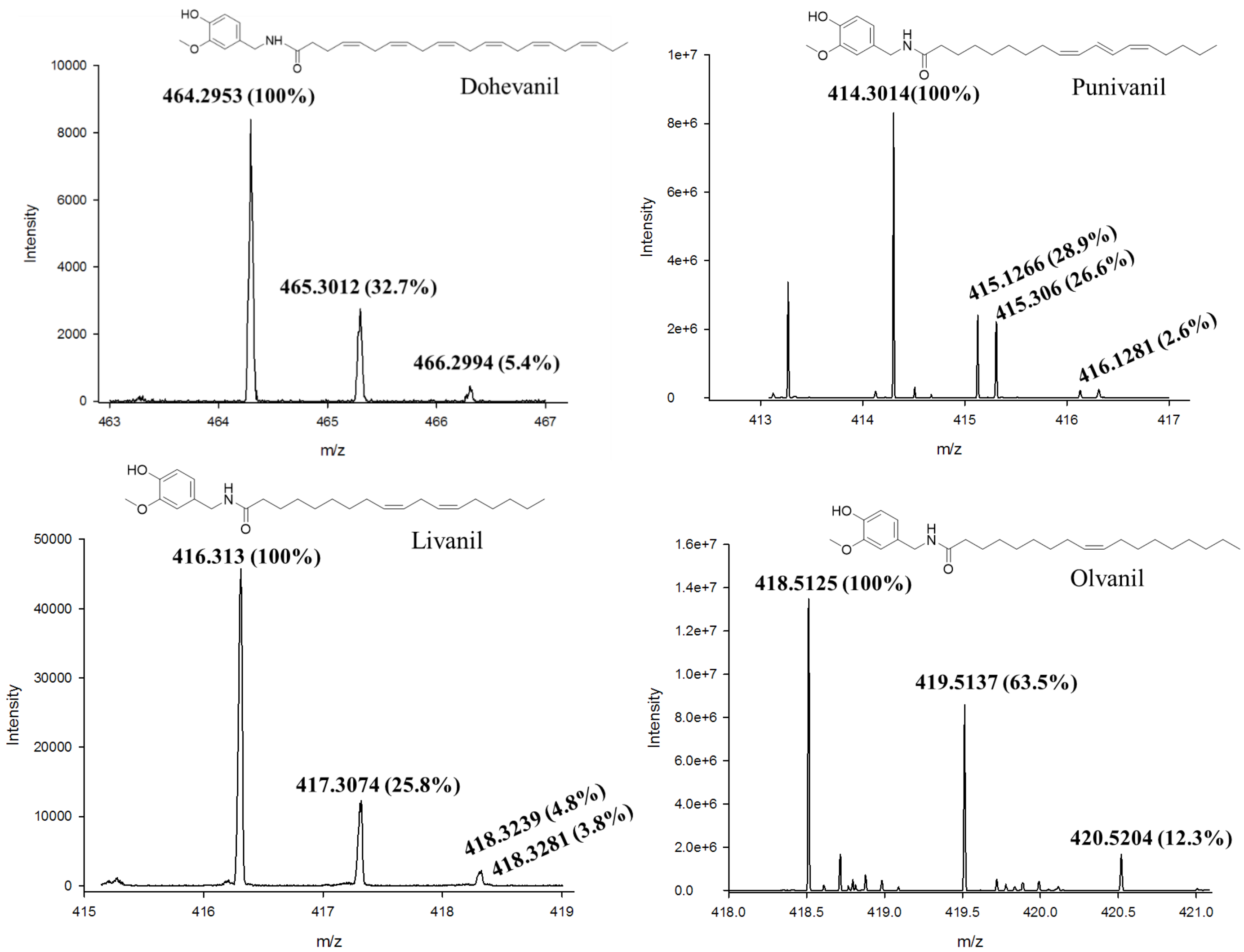

Figure S1. Ion chromatographs of N-CAPS synthetized by CALB-CLEAs obtained with QTOF/MS in positive mode (ESI+). From left to right, up to down: dohevanil, punivanil, livanil, and olvanil.

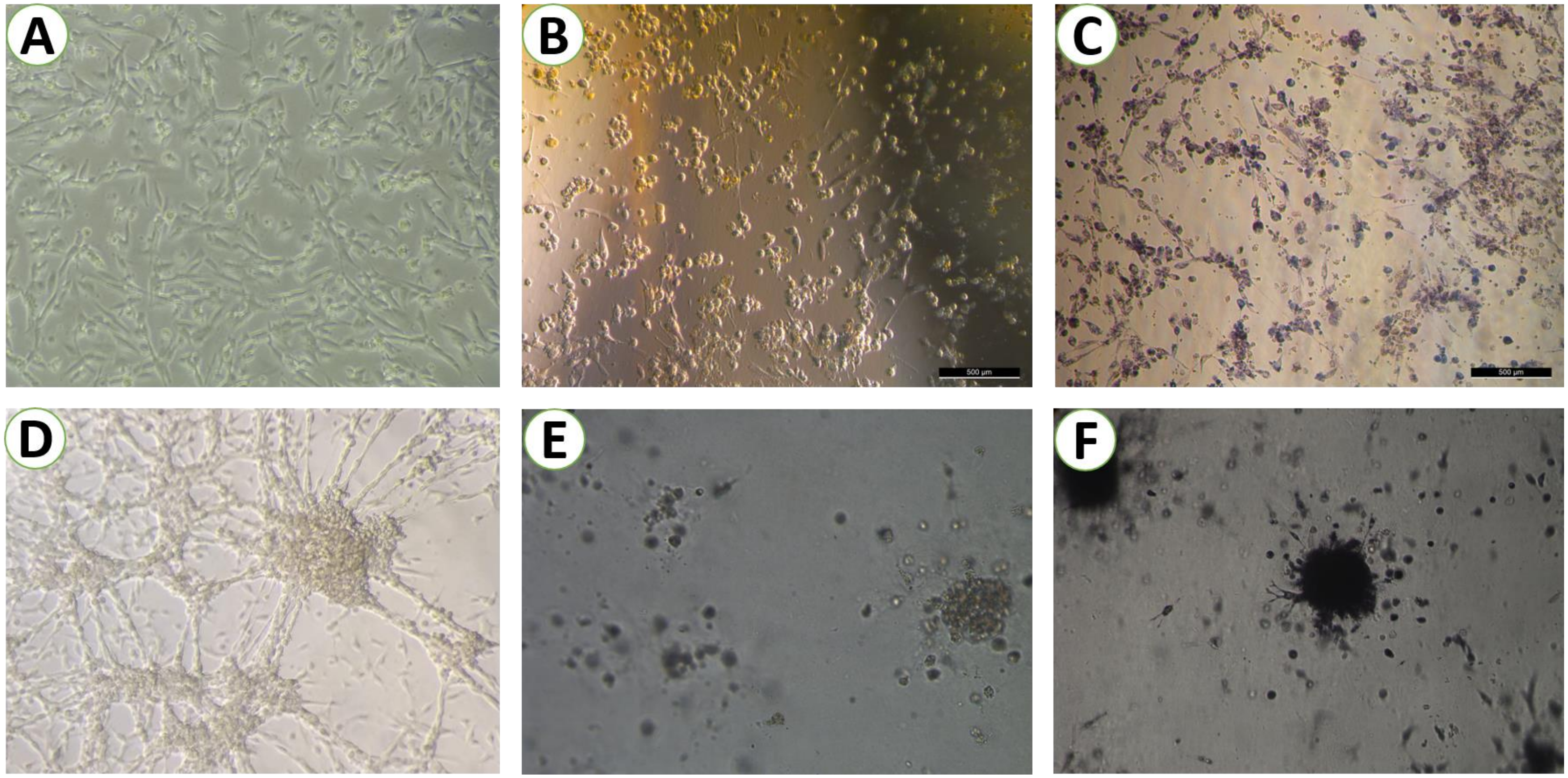

Figure S2. Photomicrographs of viable and non-viable cell lines due to capsaicinoid exposition. A) and D) show cells U-138 and U-87, respectively, with a confluency of 80% in normal growth. B) and C) show the U-138 cell line with MTT before solubilizing the formazan crystals, where B) is the group that received Dohevanil and C) is the control group, cells without formazan crystals are observed (non-viable) and cells with formazan crystals (viable or metabolically active), respectively. Likewise, E) and F) show the U-87 cells with the MTT before solubilizing the formazan crystals, E) is the group that received Dohevanil, no viability is shown, and F) is the control group, where they are shown. cells with formazan crystals (viable or metabolically active). In B) and E), morphologically round cells are shown, without their normal structure (elongated), this is an indication of non-viability.

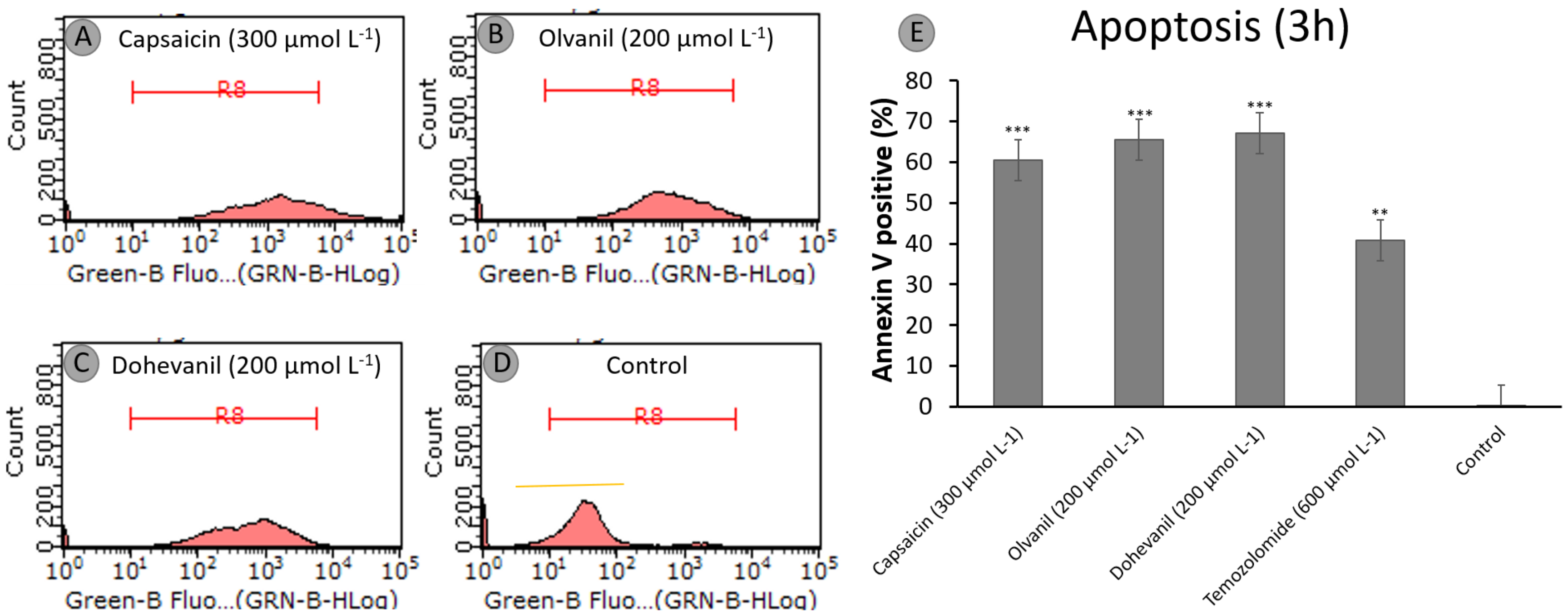

Figure S3. Induction of cell death by Apoptosis due to the effect of capsaicinoids. Percentage yielded from flow cytometry of Annexin V and PI, to determine apoptosis in GBM U-138 monolayer culture cells exposed to capsaicinoids for 6 h. A) Capsaicin (300 µmol L^{-1}), B) Olvanil (200 µmol L^{-1}), C) Dohevanil (200 µmol L^{-1}), D) Control group and E) Expressed percentage of Annexin V with the different study groups, in triplicate and analyzed using one-way ANOVA test followed by Tukey post-hoc. Differences were considered significant when $^{**}p \leq 0.001$, $^{***}p \leq 0.0001$.

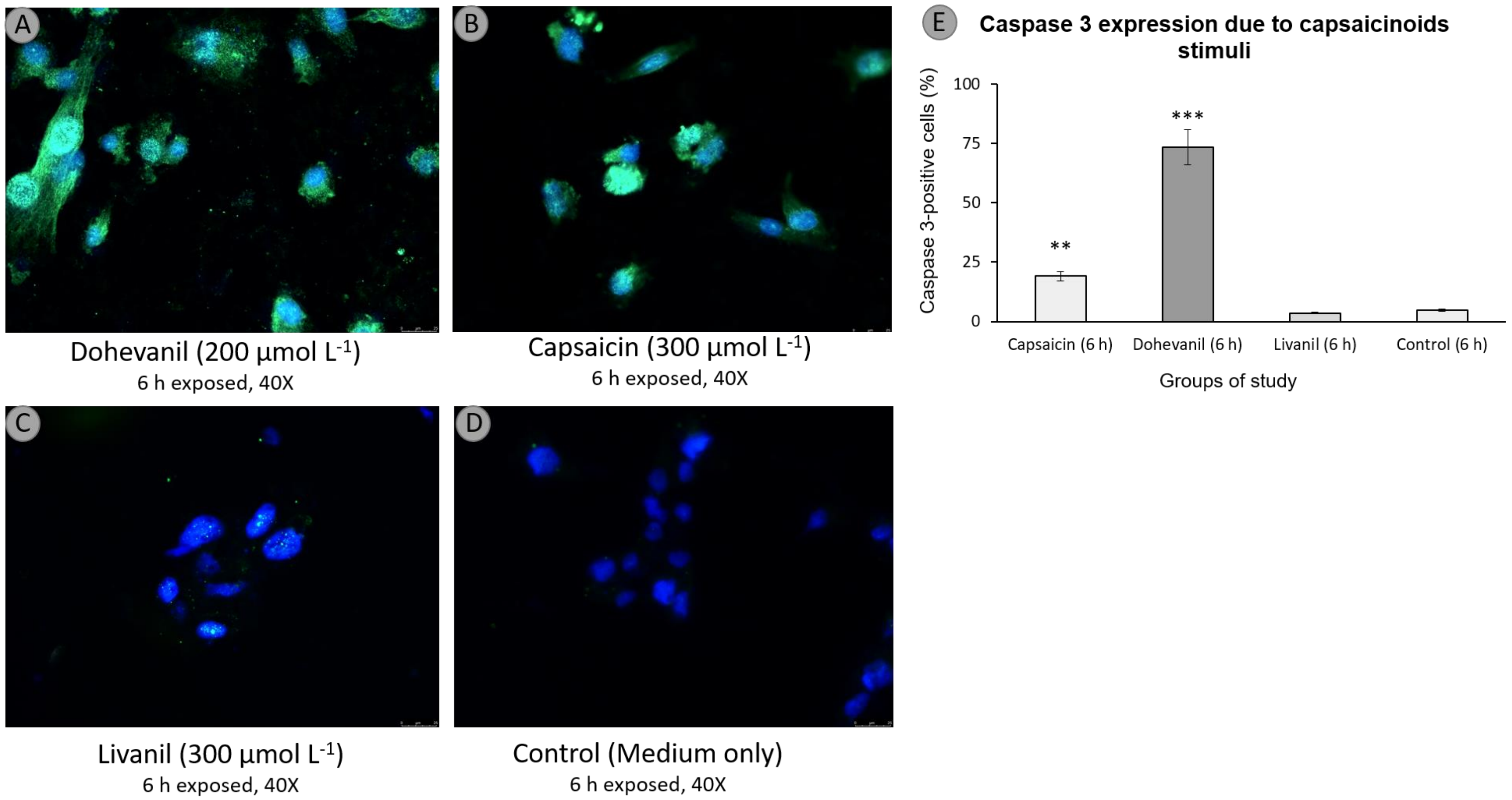

Figure S4. Expression of active caspase 3 in response to the effect of capsaicinoids by immunofluorescence of active caspase 3 in GBM U-138 cells in 3D culture, exposed for 6 h (green). DAPI labeling cell nuclei (blue). A) Dohevanil (200 µmol L^{-1}), B) Capsaicin (300 µmol L^{-1}), C) Livanil (300 µmol L^{-1}), D) Control (Images acquired at 40X). E) Percentage of cells labeled for caspase 3, of a total of 30 fields counted for each group of capsaicinoid. The analysis was performed using one-way ANOVA test followed by Tukey post-hoc. Differences were considered significant when $**p \leq 0.001$, $***p \leq 0.0001$.

cDNA and amino acid sequence of CALB Sequence of *C. antarctica* (LF 058) gene for lipase B GenBank: Z30645.1

ATGAAGCTACTCTCTCTGACCGGTGTGGCTGGTGTGCTTGCGACTTGCGTTGCAGCCACTCCTTTGGTGAAGCGTCTACCTTCCGGTTCGGACCCTGCCTTTTCGCAGCCCA
AGTCGGTGCTCGATGCGGGTCTGACCTGCCAGGGTGCTTCGCCATCCTCGGTCTCCAAACCCATCCTTCTCGTCCCCGGAACCGGCACCACAGGTCCACAGTCGTTCGACTC
GAACTGGATCCCCCTCTCAACGCAGTTGGGTTACACACCCTGCTGGATCTCACCCCCGCCGTTCATGCTCAACGACACCCAGGTCAACACGGAGTACATGGTCAACGCCAT
CACCGCGCTCTACGCTGGTTCGGGCAACAACAAGCTTCCCGTGCTTACCTGGTCCCAGGGTGGTCTGGTTGCACAGTGGGGTCTGACCTTCTTCCCCAGTATCAGGTCCAA
GGTCGATCGACTTATGGCCTTTGCGCCCGACTACAAGGGCACCGTCCTCGCCGGCCCTCTCGATGCACTCGCGGTTAGTGCACCCTCCGTATGGCAGCAAACCACCGGTTC
GGCACTCACCACCGCACTCCGAAACGCAGGTGGTCTGACCCAGATCGTGCCCACCACCAACCTCTACTCGGCGACCGACGAGATCGTTCAGCCTCAGGTGTCCAACTCGCC
ACTCGACTCATCCTACCTCTTCAACGGAAAGAACGTCCAGGCACAGGCCGTGTGTGGGCCGCTGTTCGTCATCGACCATGCAGGCTCGCTCACCTCGCAGTTCTCCTACGTC
GTCGGTCGATCCGCCCTGCGCTCCACCACGGGCCAGGCTCGTAGTGCAGACTATGGCATTACGGACTGCAACCCTCTTCCCGCCAATGATCTGACTCCCGAGCAAAAGGTC
GCCGCGGCTGCGCTCCTGGCGCCGGCAGCTGCAGCCATCGTGGCGGGTCCAAAGCAGAACTGCGAGCCCGACCTCATGCCCTACGCCCGCCCCTTTGCAGTAGGCAAAAG
GACCTGCTCCGGCATCGTCACCCCCTGA

Amino acid sequence of *C. antarctica* (LF 058) for lipase B GenBank: Z30645.1

MKLLSLTGVAGVLATCVAATPLVKRLPSGSDPAFSQPKSVLDAGLTCQGASPSSVSKPILLVPGTGTTGPQSFDSNWIPLSTQLGYTPCWISPPPFMLNDTQVNTEYMVNAITALY
AGSGNNKLPVLTWSQGGLVAQWGLTFFPSIRSKVDRLMAFAPDYKGTVLAGPLDALAVSAPSVWQQTTGSALTTALRNAGGLTQIVPTTNLYSATDEIVQPQVSNSPLDSSYLF
NGKNVQAQAVCGPLFVIDHAGSLTSQFSYVVGRSALRSTTGQARSADYGITDCNPLPANDLTPEQKVAAAALLAPAAAAIVAGPKQNCEPDLMPYARPFAVGKRTCSGIVTP*

Sequence of *C. antarctica* gene lipase B optimized by Genscript® deposited in Genbank: OR227685

ATGAAGTTGTTGAGTTTGACTGGTGTTGCCGGTGTCCTTGCTACCTGTGTCGCCGCTACCCCTTTGGTTAAGAGATTGCCTAGTGGTTCCGATCCTGCTTTTAGTCAACCAAA
GTCTGTTTTGGACGCCGGTCTTACTTGTCAGGGAGCAAGTCCATCTTCCGTTTCTAAACCTATTTTGCTTGTCCCAGGTACTGGAACTACAGGTCCTCAATCATTTGATAGTA
ACTGGATTCCATTGTCCACTCAGCTTGGATACACACCTTGCTGGATCTCACCACCTCCATTCATGTTGAACGACACACAAGTTAATACCGAATACATGGTCAATGCAATTACT
GCTTTGTATGCCGGTAGTGGAAACAATAAGTTGCCTGTTCTTACTTGGTCTCAAGGTGGATTGGTCGCTCAGTGGGGTCTTACATTTTTCCCATCTATCAGATCCAAGGTTG
ATAGATTGATGGCATTTGCTCCTGACTATAAAGGTACTGTCTTGGCAGGACCATTGGATGCCCTTGCAGTTTCAGCCCCTAGTGTCTGGCAACAGACCACTGGTTCCGCCTT
GACAACCGCACTTAGAAACGCTGGTGGATTGACACAAATTGTTCCAACTACAAATCTTTACTCAGCTACCGATGAGATCGTTCAACCTCAGGTCTCTAACTCCCCATTGGAC
TCAAGTTATCTTTTCAACGGTAAAAATGTTCAAGCTCAGGCCGTCTGTGGTCCTTTGTTTGTTATTGATCATGCTGGATCTTTGACTTCCCAATTCTCATACGTTGTCGGAAG
ATCCGCTTTGAGATCAACCACTGGTCAGGCAAGATCTGCTGATTATGGAATTACCGACTGTAACCCTTTGCCAGCTAATGATCTTACTCCAGAACAAAAGGTTGCTGCCGCA
GCTTTGCTTGCTCCTGCCGCAGCTGCCATCGTTGCCGGTCCTAAACAAAATTGCGAGCCAGACTTGATGCCTTACGCAAGACCATTCGCAGTCGGAAAAAGAACATGCTCA
GGTATTGTCACTCCATAA

Amino acid sequence of *C. antarctica* lipase B optimized by Genscript® deposited in Genbank: OR227685

MKLLSLTGVAGVLATCVAATPLVKRLPSGSDPAFSQPKSVLDAGLTCQGASPSSVSKPILLVPGTGTTGPQSFDSNWIPLSTQLGYTPCWISPPPFMLNDTQVNTEYMVNAITALY
AGSGNNKLPVLTWSQGGLVAQWGLTFFPSIRSKVDRLMAFAPDYKGTVLAGPLDALAVSAPSVWQQTTGSALTTALRNAGGLTQIVPTTNLYSATDEIVQPQVSNSPLDSSYLF
NGKNVQAQAVCGPLFVIDHAGSLTSQFSYVVGRSALRSTTGQARSADYGITDCNPLPANDLTPEQKVAAAALLAPAAAAIVAGPKQNCEPDLMPYARPFAVGKRTCSGIVTP*
